# Individual variability in subcortical neural encoding shapes phonetic cue weighting

**DOI:** 10.1038/s41598-023-37212-y

**Published:** 2023-06-20

**Authors:** Jinghua Ou, Ming Xiang, Alan C. L. Yu

**Affiliations:** Department of Linguistics, University of Chicago, 1115 E. 58Th Street, Chicago, IL 60637 USA

**Keywords:** Cognitive neuroscience, Human behaviour

## Abstract

Recent studies have revealed great individual variability in cue weighting, and such variation is shown to be systematic across individuals and linked to differences in some general cognitive mechanism. The present study investigated the role of subcortical encoding as a source of individual variability in cue weighting by focusing on English listeners’ frequency following responses to the tense/lax English vowel contrast varying in spectral and durational cues. Listeners differed in early auditory encoding with some encoding the spectral cue more veridically than the durational one, while others exhibited the reverse pattern. These differences in cue encoding further correlate with behavioral variability in cue weighting, suggesting that specificity in cue encoding across individuals modulates how cues are weighted in downstream processes.

## Introduction

To understand speech, listeners need to perceptually organize acoustic cues (i.e., the spectrotemporal components of the speech signal) into coherent phonetically coded speech percepts. These acoustic cues are weighted differently by listeners depending on the linguistic contrast in a particular language^1–3^. For example, the tense-lax vowel contrast in English (e.g., /i/ vs. /ɪ/) is cued by both spectral differences and duration differences, but spectral information is more important than the durational one^4–6^. Recent studies have revealed great individual variability in cue weighting^7–9^, and such variation has been shown to be systematic across contrasts^10,11^ and across time^12^. Factors governing such variations remain largely under-explored, however. The present study aims to examine whether individual variability in cue weighting might stem from differences in early stage of auditory encoding by measuring listeners’ frequency-following response (FFR).

Variability in cue weighting is driven by differences in individual perceptual experience, such as L1 background^1,13^ or L2 exposure^14,15^. However, even if listeners consistently weight one cue more than the other(s), significant individual variation can still be observed in weight between cues. For example, Kong and Edwards^8^ found significant individual variations in the use of fundamental frequency (F0) for the stop initial voicing distinction in English, even though listeners consistently weighed voice onset time (VOT) more than F0. An experience-driven account is thus insufficient to explain why the relative weighting between cues is not uniform across listeners, given that these listeners shared similar language background. This observation led to the hypothesis that such variation might at least partially stem from differences in some general cognitive mechanisms that modulate cue weighting. Kapnoula et al.^7^, for example, linked individual variation in cue weighting between VOT and F0 to categorization gradience measured in a visual analogue scaling (VAS) task (i.e., a task that asks the listener to rate stimuli along a visually presented continuous scale, such a line, ranging from one category, say a perfect /b/, to another, say a perfect /p/), such that listeners who exhibited a more gradient categorization pattern (i.e., a more continuous response distribution along the scale) are more likely to assign more weight to the secondary F0 cue. Differential cue weighting might also arise from differences in processing strategies. Ou et al.^11^ examined secondary cue weighting in two sets of English contrasts (/b/ vs. /p/ and /i/ vs. /ɪ/) using an eye-tracking paradigm and found that listeners who assigned more weights to secondary cues tend to adopt a buffer processing strategy where acoustic cues are stored in a memory buffer until all relevant cues are available. Alternatively, those who assigned less weights to secondary cue tend to use a continuous cascading processing strategy such that relevant cue information is taken into account as soon as they become available.

Individual differences in these cognitive mechanisms may ultimately stem from differences in an earlier stage of speech processing such as in the neural encoding of the acoustic cues. For instance, categorization gradience as measured in a VAS task with voicing contrast varying in VOT and F0 has been shown to be associated with gradient cortical speech representation^16^. In that study, individuals with low gradience (i.e., responses clustering around two ends of the scale) showed a step-like response pattern of N1, a negative event-related-potential (ERP) component reflecting initial formation of object representation in early auditory cortex^17^, while a linear relationship between N1 amplitude and VOTs was observed among listeners with high gradience. However, the source of the cortical N1 difference is unclear, as acoustic information undergoes several layers of neural processing prior to the signal arriving at the cortical level. Recent studies have revealed that differences in categorization are subserved by how cues are encoded at the subcortical level. For instance, the latency of subcortical responses to the voicing onset predicts the VOT categorization differences in a 2-alternative-forced-choice (2AFC) task between English and Spanish speakers^18^. Additionally, there is also evidence that the uncertainty in VOT categorization in a 2AFC task among English speakers correlates with how faithfully subcortical responses encode VOT differences, with listeners who showed high categorization uncertainty (i.e., shallower slopes of the classification function) exhibiting less faithful encoding of the acoustic differences^19^. Listeners with high categorization uncertainty exhibit a more linear encoding of acoustic differences at the cortical level, whereas listeners with low categorization uncertainty exhibited N1 responses that reflect a more discrete category pattern. These studies primarily focus on neural encoding of a speech continuum varying in one acoustic dimension. It is not clear if variability in the subcortical encoding of one cue dimension would necessarily map onto variability of encoding of other cue dimensions. A notable exception is Tamura and Sung^20^, which examined subcortical and cortical responses to a /da/-/ta/ continuum varying in VOT and F0 from native Japanese and Korean listeners to investigate the differences in cue weighting for voicing distinction. Behaviourally, Japanese speakers assigned more weights to VOT than F0, while the reversed pattern was observed for the Korean speakers. Yet, they found that the subcortical encoding of VOT did not differ significantly between the two groups of listeners, even though the Japanese listeners’ cortical N1 responses were much more sensitive to the VOT distinction than those of the Korean listeners. While they concluded the processing of acoustic cue weighting in phonetic perception is reflected in the early cortical auditory activity and not in the subcortical activities, their findings only account for cross-linguistic differences. It remains unclear if different cue dimensions are differentially encoded within the same individual and if such differences are related to individual differences in cue weighting.

Given that secondary cue weighting is associated with categorization gradience^7^ and uncertainty^11^, and categorization uncertainty in turn is associated with the quality of subcortical encoding^19^, the present study aims to elucidate the role of subcortical encoding as a potential source of individual variability in cue weighting by focusing on English listeners’ subcortical responses to the tense/lax vowel contrast (/i/ vs. /ɪ/), which is primarily cued by spectral (Formant) and durational (Vowel Duration) cues. Specifically, subcortical FFRs were measured to assess the precision with which individuals encode the speech stimuli. The FFR is part of the speech-evoked subcortical response and reflects the encoding of sustained periodic information (i.e., fundamental frequency in tones and higher harmonics in sonorant sounds such as vowels). Because FFRs are rich in temporal and spectral information, we can use multiple indices to examine subcortical encoding in a holistic manner; and we can also dissect individual components of the FFR response to examine how they reflect distinct aspects of the speech stimuli. Two hypotheses were tested. First, individuals who demonstrate relatively higher secondary cue weight may also have better general auditory encoding ability. Their encoding ability affords more robust encoding of the secondary cue and subsequently deeper integration of such information (Hypothesis 1). We evaluated this hypothesis by constructing a global measure of stimulus-to-response correlations (SRC) between subcortical responses and stimulus waveforms, which indicates how closely the FFR mirrors the overall stimulus waveform without making reference to any specific cue. On the other hand, individual variability in cue weighting might reflect cue-specific differences in encoding (Hypothesis 2). To this end, we measured, Formant and Vowel Duration (VD) encoding separately. If the two cues were differentially encoded in the FFRs with one cue better encoded than the other, it might facilitate the weighting of one cue over the other in the downstream process.

## Results

Vowel categorization was examined in a 2AFC task to derive weights of each cue, and a behavioral cue-weight ratio was computed to index the relative weighting of cues within individuals (see “Behavioral data analysis” for details). To test Hypothesis 1, the FFRs elicited by the vowel continuum were analyzed holistically. Specifically, we calculated the global measure of SRC to indicate the overall quality of the FFRs. To test Hypothesis 2, we analyzed the FFRs in relation to specific acoustic features, namely the Formant and VD cues (see “EEG data analysis” for details). These two different sets of neural indices were then used to predict the behavioral cue-weight ratios.

### Behavioral cue weighting

**Figure 1:**
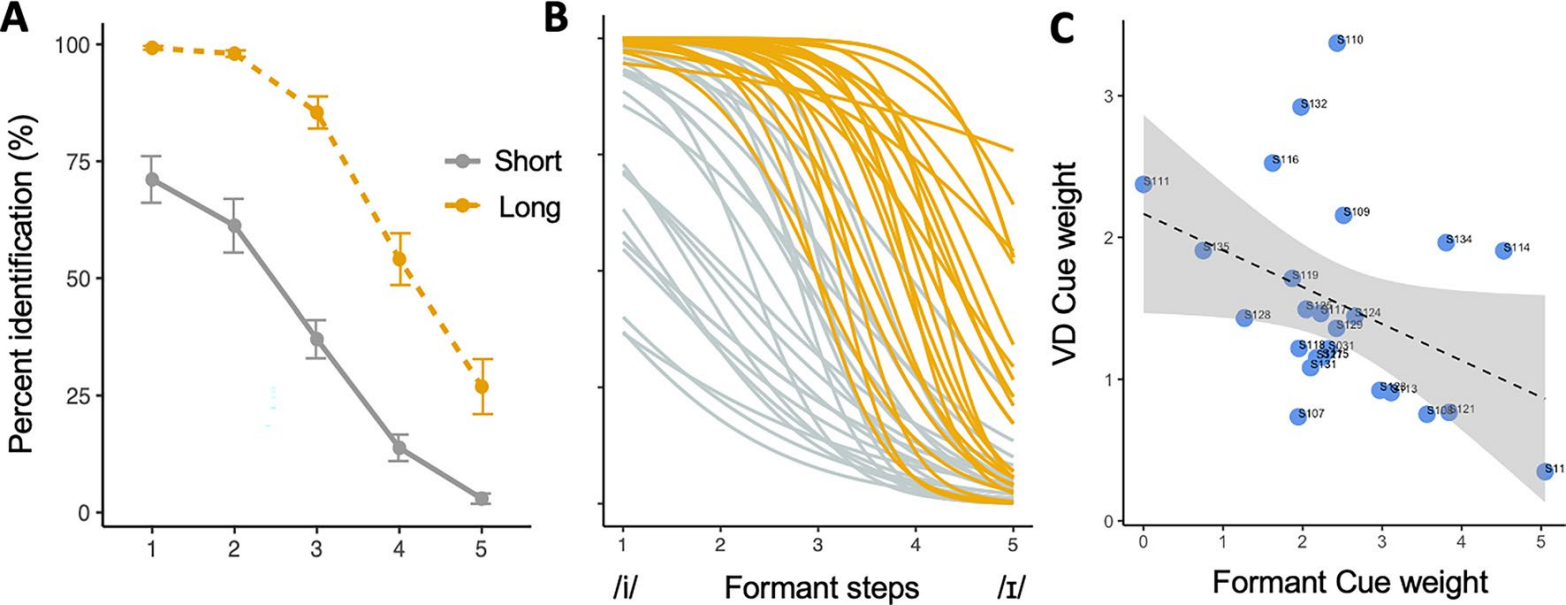
**(A)** Mean identification response of /i/ along the vowel continuum with short (in grey) and long VD (in yellow) averaged across participants. Error bars represent ± 1 s.e.m. (**B)** Identification responses from each participant. (**C**) Correlation between Formant and Vowel Duration (VD) cue weights. Dotted lines indicate linear functions with shaded regions showing 95% confidence interval.

In the behavioral categorization task, participants classified sounds drawn randomly from a “deep” ‒ “dip” continuum with five steps of formant frequency and two steps of VDs. The mean identification responses for the vowel continuum are shown in Fig. 1A. Results of a mixed-effect logistic regression showed that both Formant [β = − 1.77, z = − 28.85, *p* < 0.001] and VD [β = 1.25, z = 22.25, *p* < 0.001] were significant predictors of listener response patterns at the group level. The effect of Formant was greater than that of VD (β_Formant_ = 1.77 vs. β_VD_ = 1.25), confirming the status of Formant as more important for differentiating the contrast between /i/ and /ɪ/. Moreover, the individual Formant and VD cue weight showed a negative correlation (*r* = − 0.39, *p* = 0.05) suggesting a trading relationship between the two (Fig. 1C). Individual response patterns (Fig. 1B) revealed that subject’s mean rate of /i/-identification response was widely dispersed between 0 and 100%, suggesting that individual listeners weighted the two cues differently.

### Neural cue encoding

FFRs were analyzed with respects to the two hypotheses. To test Hypothesis 1, we computed the SRC to indicate how closely the FFR waveforms mirror the vowel segment of the stimulus without making reference to any specific cue. To test Hypothesis 2, the fast Fourier transform (FFT) was used to track Formant information as encoded in the FFR^21,22^, and VD was measured by estimating the time-course of periodicity using autocorrelation^23^. These formant/VD tracking measures of FFR were used to correlate with the acoustic stimulus values to index how closely the tracking follows the auditory input^19,21^ that is, F1-encoding and VD-encoding indices (see EEG data analysis for a detail description on how the two sets of neural encoding indices were constructed).

**Figure 2:**
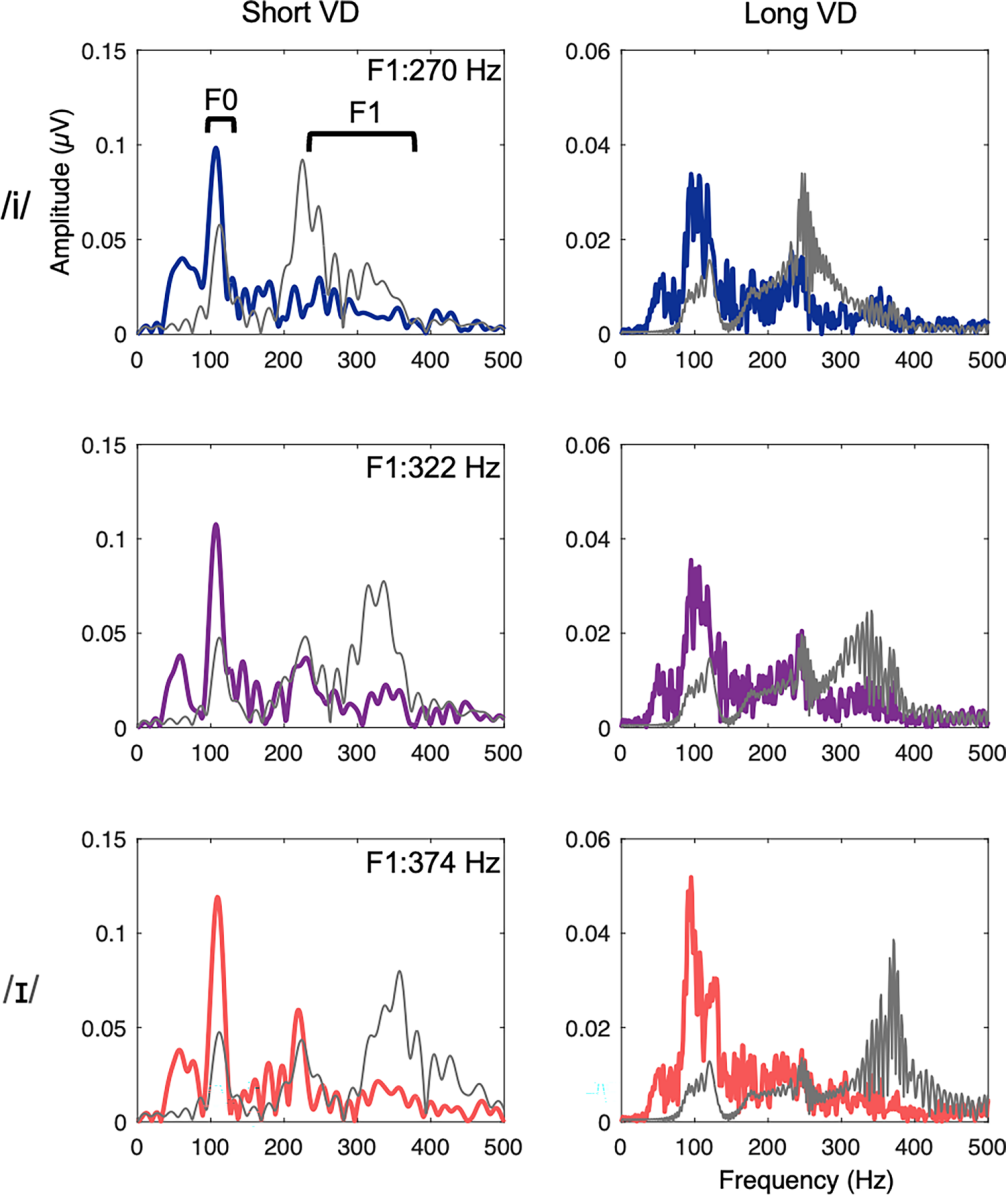
Spectral representations of the group-averaged FFR by three Formant (in rows) and two VD (in columns) conditions via FFT, overlaid by the stimulus FFTs (in grey).

The salience of the first Formant (F1) contained in the FFRs was used as a proxy for the encoding of the Formant structures in the stimuli. Figure 2 shows clear energy peaks in the F0 range (100 to 125 Hz) and in the F1 range (200 to 400 Hz). F1-encoding estimated from the FFTs based on group-averaged FFRs were 282 Hz, 338 Hz, and 367 Hz respectively, which closely paralleled changes in F1 along the stimulus continuum. For each participant, automated peak selection in the F1 frequency range was performed using custom routines coded in MATLAB and visually confirmed by referring to the individual FFTs.

**Figure 3:**
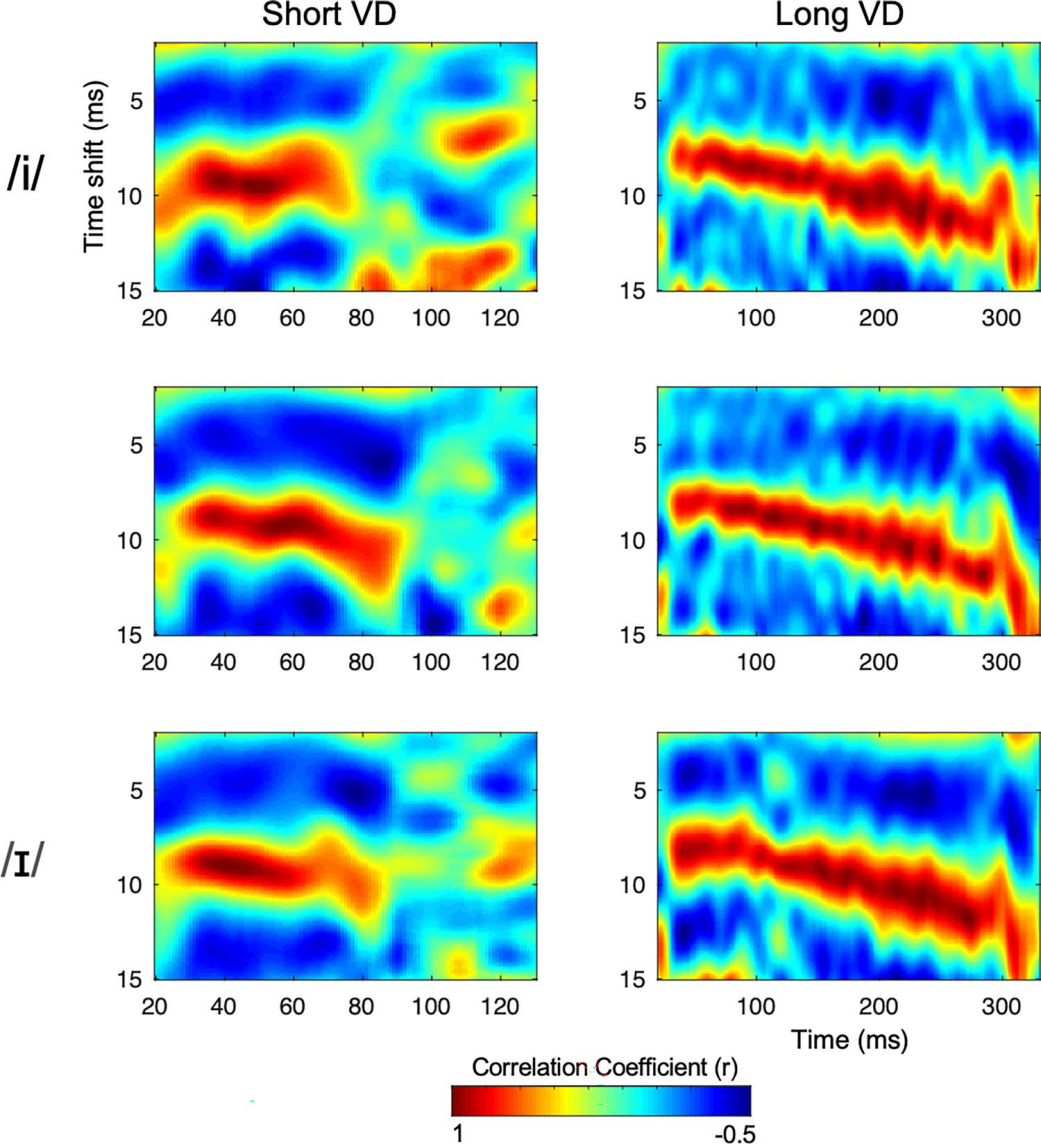
Autocorrelogram of the FFRs by three Formant (in rows) and two VD (in columns) conditions. Colors represent the strength of correlation, with warmer color indicating higher correlation. The warm bands of color closely follow the inverse of the F0 (frequency = 1/time shift).

Vowel duration was estimated by how well the FFR tracked the time-course of fundamental frequency (F0). Figure 3 demonstrates the autocorrelograms based on the group-averaged FFRs. In each stimulus condition, we observed robust phase locking at around 10 ms time shift, reciprocal of which corresponds to the F0 of the stimuli (i.e., 100 Hz; frequency = 1/time shift). The VD-encoding estimated from the group-averaged autocorrelation were 44 ms and 229 ms respectively. For each participant, automated selection of onset and offset time bin and computation of onset-offset time differences were performed using routines coded in MATLAB.

### Brain-behavior relations

The behavioral Formant and VD cue weights were obtained based on cue coefficients derived from logistic regression model. To examine the brain-behavior relations, the cue-specific neural encoding indices were used to correlate with the behavioral cue weights, and the results show a strong correlation between F1-encoding and Formant cue weights (*r* = 0.61, *p* < 0.01), and moderate correlation between VD-encoding and VD cue weight (*r* = 0.39, *p* = 0.04). While the correlations between cue-specific neural encoding indices and perceptual cue weights demonstrated that, as a group, listeners rely more on F1 than VD, which is consistent with the behavioral findings in the present study, these findings do not provide any information regarding the relative importance of cues within individuals. A measure that directly compares the relative importance between perceptual cue weights on the one hand and the relative strength of the neural encoding on the other is required. To this end, we computed a cue-weight ratio by dividing a participant’s VD cue weight by that participant’s Formant cue weight, with larger values corresponding to more reliance on the VD cue. Similarly, to compare the relative encoding quality of each cue within an individual, a cue-encoding ratio was computed by diving a participant’s VD-encoding index by that participant’s F1-encoding index. The two neural indices, namely cue-encoding ratio and SRC, were used to predict individual differences in perceptual cue weighting. We constructed a multiple regression model with the behavioral index of cue weighting (i.e., behavioral cue-weight ratio) as the dependent variable, and the two neural indices (i.e., cue-encoding ratio and SRC) as the fixed effects.

**Figure 4:**
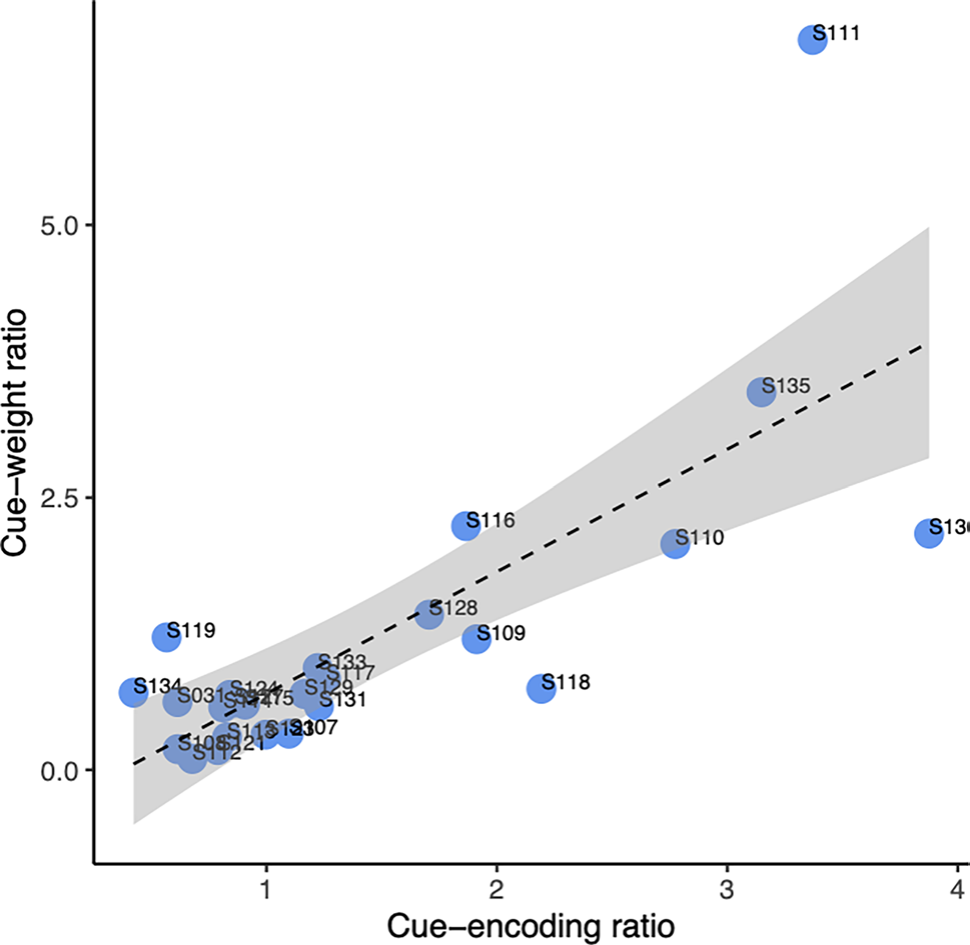
Scatterplots behavioral cue-weight ratio and neural cue-encoding ratio. Dotted lines indicate linear functions between the two measures with shaded regions showing 95% confidence interval. The subject number is shown on each data point.

Results of the regression model showed a significant effect of cue-encoding ratio [β = 1.07, t = 5.17, *p* < 0.001] but no effect of SRC [β = − 1.26, t = − 0.68, *p* = 0.50] (Table 1). Specifically, the cue-encoding ratio showed a positive relationship with behavioral cue-weight ratio (Fig. 4), suggesting higher neural VD-F1 ratios associated with higher behavioral VD-F1 ratios. That is, listeners who encoded VD more veridically than F1 in the FFR also weighted the VD cue more strongly than F1 during vowel categorization.

**Table 1:** Results of the regression model for cue-weight ratio that included stimulus-to-response correlation (SRC) and cue-encoding ratio as fixed effects.

Predictor	β	*SE*	*t*	*p*
Intercept	0.17	0.93	0.18	.853
SRC	− 1.26	1.85	− 0.68	.500
Cue-encoding ratio	1.08	0.21	5.17	< .001***

*** p < 0.001

We constructed another regression model that included cue-encoding ratio as well as years of musical training and self-rated L2 proficiency as fixed factors, to evaluate the potential effects of musical training and L2 proficiency on cue weighting. Results showed that musical [β = − 0.03, t = − 0.66, *p* = 0.51] and L2 experience [β = − 0.09, t = − 0.92, *p* = 0.36] did not have a significant effect on the cue-weight ratio, and the cue-encoding ratio remained as the significant predictor [β = 1.09, t = 5.45, *p* < 0.001] (Appendix Table S3). These results suggest that the subject’s musical and L2 experiences had limited influence on how a listener weight speech cues as compared to neural encoding in the current study.

## Discussion

The present study investigated whether and how individual subcortical encoding of the acoustic signals influences how cues were weighted when categorizing an English vowel contrast varying orthogonally in two acoustic dimensions (i.e., Formant and VD). We observed that the two acoustic cues were differentially encoded at the subcortical level across individuals, with some encoding VD better than Formant (or vice versa) while some showed equal encoding of the two. Crucially, not only are the cue-specific neural encoding indices correlated significantly with perceptual cue weights, the differential encoding as indexed by the cue-encoding ratio is associated with the cue-weight ratio from the identification task, suggesting that individual subcortical encoding as a source for differences in cue weighting. Another important finding is that cue-specific encoding, rather than general encoding ability modulates how cues are weighted in downstream process. This finding is in contrast with the findings from Tamura and Sung^20^ concerning cross-linguistic differences in cue weight. They did not observe any general or cue-specific differences in subcortical activities, even though the two languages differ in their weighting of VOT and F0 cues. The fact that individual listeners who exhibit differences in perceptual cue weights show corresponding cue-specific differences in neural encoding suggests that individual differences in cue weighting is likely to be driven by fundamental neurocognitive differences across individuals. At the cross-linguistic level, differences in perceptual cue weights might be linked to the mapping of subcortical information to cortical processes (as suggested by N1 differences to VOT differences between Japanese and Korean listeners), rather than population-wide differences in subcortical encoding differences. To be sure, our findings cannot rule out past linguistic experiences as a potential factor in contributing to individual differences in cue weighting, particularly since subcortical response activities have been shown to be sensitive to training effects^24^. However, since studies on the acoustic realization of the tense-lax difference in English have repeatedly found the primary acoustic cue to be spectral difference^4–6^, it does not seem likely that language experience alone is sufficient to account for the individual variability.

The FFR results showed that listeners differed in early auditory neural encoding with some encoding the spectral cue (i.e., Formant) more veridically than the temporal cue (i.e., VD), while others exhibited the reverse pattern, suggesting specificity in cue encoding across individuals. These individual differences in cue encoding echoes the different perceptual patterns observed in perception of missing fundamental tones^25,26^, in that some individuals tend to identify the pitch of such tones with the missing F0 (“F0 listeners”) while others based their judgment on the frequency of the partials that make up the tones (“spectral listeners”). These perceptual preferences have been tied to differences in neuroanatomy, specifically to differences in the cortical volume of the Heschl’s gyrus (a pitch detection area) in the left and right hemisphere^27^. Such a spectral vs. temporal cue dependence is even more apparent among listeners with cochlear implant (CI), who consistently show decreased use of spectral cues and greater use of durational cues, due to reduced spectral resolution of the auditory system^28–30^. In the present study, we found different patterns of weighting the spectral and temporal cues in vowel categorization, and these weighting differences in turn depend on how spectrotemporal information is encoded at the earliest stage of processing in the auditory system. To be sure, whether the association between perceptual cue weight and subcortical tracking of the corresponding acoustic cue generalizes to other phonetic cues remains an empirical question that will require further investigation, the association is not likely to be unique to cues that are in a trading relationship as in the present case. After all, most, if not all, phonetic contrasts are supported by multiple cue dimensions. Such cues may not be related to each other at all, or, if they were, they may be positively or negatively correlated^10^.

One caveat about the present study worth highlighting is the fact that different methods were used to measure the neural representation of each cue (i.e., FFT to track Formant and autocorrelation for the VD cues). We chose to use different methods for Formant and VD tracking based on the strengths and limitations of each method. FFT is a common method for computing spectral composition of a signal but provides no information about time-varying frequency content. While there are variants of FFT, such as the Short-Time Fourier Transform (STFT)^31,32^ and wavelet transform^33^, which can be used to track spectral frequency over time, these methods have a trade-off between time and frequency resolution and may not provide the most accurate estimation for both cue dimensions. Autocorrelation was used to complement the FFT to accurately capture the temporal information of the signal. We believe that utilizing these two different methods allows us to best capture the different aspects of the neural encoding that may contribute to phonetic cue weighting. To be sure, the use of different methods to measure neural representation may limit the direct comparison between the two due to their distinct underlying computations. To mitigate such concerns, our approach—correlating the tracking values with acoustic values before comparing the two correlation measures through a ratio ‒ allows us to compare cue encoding at the level of similarity, rather than at the level of the original signal patterns, thus ensuring comparability between cue dimensions. Nevertheless, to seek a secondary confirmation of the current findings, we performed Time Frequency Analysis (TFA) to track spectral frequency over time of the neural signals, and thus Formant and VD cue encoding were extracted using the same computational method (see Supplementary analysis for details). The TFA results reinforced the positive correlation between cue-encoding ratio and cue-weight ratio (*r* = 0.68, *p* < 0.001), even if the magnitude the correlational strength using TFA was slightly weaker compared to the correlation obtained using the FFT and autocorrelation methods (*r* = 0.78); the weaker TFA-based correlation might stem from the lower spectral resolution of the TFA.

Our findings do not support a lack of sensitivity to the acoustic details as a general explanation for individual variability in cue weighting. While previous studies have observed a relationship between auditory encoding and perceptual performance^19,34,35^, these studies have focused on stimuli that varying in one acoustic dimension. By employing stimuli varying in two acoustic dimensions as well as comprehensively evaluating the encoding of the two cues, our study found that the relationship between auditory encoding and perceptual performance is cue-specific and can vary across individuals. Furthermore, while previous studies have found that individual differences in perceptual cue weighting might stem from individual general cognitive mechanisms^7,11^, our study identifies the central role of auditory cue encoding in explaining perceptual cue weight differences. To be sure, the mechanisms of categorization gradience, processing strategy, as well as auditory encoding might all play a role in shaping variability in cue weighting, especially since they operate at different stages of speech processing. Whether and how these mechanisms operate within the same individuals merits further investigation.

To summarize, by demonstrating that listeners show differential cue encoding, which then affects the reliability and weighting of certain cues that support phonological contrasts, the present study sheds important light on the mechanisms underlying variability in cue weighting, moreover, provides a foundation for further research to elucidate how perceptual variability might be shaped by different mechanisms at play.

## Materials and methods

### Participants

Thirty young adults participated in the experiment. Three participants were excluded due to excessive artifacts in the data (electrical noise or high levels of myogenic noise), and two additional participants were excluded due to technical issues during data recording. Final sample consisted of 25 subjects (age: 21.79 ± 3.03 years; 9 males), all were university students with no reported speech, hearing, or language disorders. Experimental procedures were approved by the Social and Behavioral Science Institutional Review Board at the University of Chicago and were in accordance with the Declaration of Helsinki. Informed written consents were obtained from all participants.

Participants completed a survey of their language learning and musical training history (Appendix Table S1). Participants started their musical training between age 3 to 12 (mean = 7.59, SD = 2.68), and spent an average of 7.25 years (SD = 4.43) in training. All participants identified English as their first language except for three, who identified an additional language, i.e., Japanese, Mandarin, and Hungarian, as first language. Self-rated second-language (L2) proficiency for speaking and listening abilities were on average 4.37 (SD = 1.24) and 3.98 (SD = 1.16) on a 7-point Likert-type scale ranging from 1 (very poor) to 7 (native-like). All participants reported that English is the dominant language in daily life. To account for the potential effects of musical and language experience on speech cue weighting, years of musical training and self-rated L2 proficiency (averaged for speaking and listening) were entered as covariates in the brain-behavior analyses.

### Stimuli

Auditory stimuli were synthesized tokens from a /i/-/ɪ/ continuum with varying Formant frequencies and VD. Stimuli were created using a Klatt style synthesizer implemented in Matlab, with formant and VD values set with reference to those of Gordon et al.^36^. A five-step formant frequency continuum were combined with two VDs (60 ms and 300 ms) resulting in a total of 10 vowel stimuli. The formant continuum was constructed by varying the center frequencies of the first three formants in equal steps from /i/ to /ɪ/ (see Appendix Table S2). The fourth and fifth formants were held constant at 3500 and 4500 Hz, respectively. The rise and decay times were set at 1/6 of the vowel duration, with 10 ms for the short vowels and 50 ms for the long vowels. The fundamental frequency (F0) in all stimuli fell linearly from 125 Hz at onset to 100 Hz at offset. Finally the vowel tokens were embedded in a /d_p/ frame to generate a /deep/-/dip/ continuum.

### Behavioral identification task

Participants completed a forced-choice identification task in an acoustically shielded booth before the electroencephalography (EEG) recording. The auditory stimuli were delivered binaurally through Sennheiser HD555 headphones at 80 dB SPL, and the experiment was controlled with E-Prime. Participants were instructed to identify the sound as quickly as possible (/deep/ or /dip/) through button presses on an E-prime response box within 2 s. Each of the ten tokens from the continuum was repeated 15 times in a randomized order for a total of 150 trials.

### EEG data acquisition and preprocessing

Six tokens from the /deep/-/dip/ continuum (i.e., Formant step 1, 3, and 5 with short and long VD) were used to elicit electrophysiological responses. Stimulus delivery was controlled in MATLAB, and delivered binaurally at an intensity of 80 dB SPL through inserted earphones (ER-1, Etymotic Research, IL). The stimuli were presented in alternating polarities for a total of 2000 sweeps per stimulus token (1000 per polarity) with an inter-stimulus interval jittered among 350, 390, 400, 410 ms. The six tokens were presented in separate blocks with block order randomized across participants. The entire EEG recording lasted about 2 h.

Electrophysiological responses were collected using the Brain Products actiCHamp, EP-preamp amplifiers and BrainVision Recorder software (Brain Products GmbH), with Ag–AgCl scalp electrodes placed at vertex (Cz, active) referenced to linked mastoids (M1/M2), and the mid-forehead as ground. Data were digitized at a sampling rate of 25 kHz, with no online filters applied. Electrode impedances were kept below 5 kΩ, and the preamplifier gain was set to 50. All recordings were made passively with the participant sitting comfortably in the booth. To minimize myogenic artifacts, participants were instructed to relax and refrain from extraneous body movement, and to ignore the stimuli as they watched a silent movie throughout the recording session.

The continuous EEG recordings were bandpass filtered off-line from 80 to 2500 Hz (12 dB/octave, zero phase-shift). The recordings were then epoched into segments that were time locked to the auditory stimuli (short VD: − 50 to 150 ms; long VD: − 50 to 350 ms). After baseline correcting each response to the mean voltage of the pre-stimulus region, trials with amplitudes exceeding a predefined range ± 35 μV were rejected. The artifact-free trials (1765 ± 95) were averaged for each stimulus condition for each subject, and downsampled from 25 to 10 k Hz.

### Behavioral data analysis

Logistic regressions were fitted to each participant’s identification of the stimulus as /deep/, with Formant, VD step and their interaction as fixed factors. Coefficients for Formant and VD were used as indices of cue weights to compute a cue-weight ratio (similar to the “reliance ratio” used by Escudero and Boersma 2004) for each participant by dividing the VD cue weight by the Formant cue weight, with a ratio of 1 or close to 1 reflecting equal weighting of both cues. A ratio larger than 1 indicates that the listener relies more on the VD cue relative to the Formant cue; on the other hand, a ratio smaller than 1 reflects more reliance on the Formant cue than the VD cue.

### EEG data analysis

Per Hypothesis 1, which attributes individual differences in perceptual cue weighting to general auditory encoding ability across listeners, we computed an index to indicate how closely the FFR waveforms mirror the vowel segment of the stimulus without making reference to any specific cue. The stimuli were downsampled to 10 kHz to match the sampling rate of the FFRs. For each FFR, we first calculated its estimated onset delay relative to the vowel onset time (neural lag) due to neural conduction of the auditory pathway. This neural lag was computed by cross correlating the FFR and the stimulus vowel waveform in time with respect to one another^37^. The neural lag value (in ms) was taken as the time point in which maximum positive correlation was achieved between 6 and 12 ms, the expected latency of the onset component of the auditory subcortical response^38^. The onset of the FFR was adjusted by the neural lag, and the vowel segment of the stimulus was truncated from the end by the length of the neural lag, so that the number of data points in the two signals were matched. The stimulus-to-response correlations (SRCs) were generated by correlating FFRs (the portion of FFR from neural lag to 60 ms/300 ms) with stimulus vowel waveforms using Pearson’s correlation. This measure represents both the strength and direction of the linear relationship between the two signals. The SRC was calculated for each stimulus, then averaged across six the stimulus conditions for each participant.

Per Hypothesis 2, which relates listener-specific perceptual cue weighting to individual differences in cue-specific neural encoding, we quantified the subcortical encoding of the two cues respectively. The F1 in the FFRs was used as a proxy for the encoding of the Formant structures in the stimuli. This is due to the phase-locking limitation of the subcortical structures, which limits neural encoding of the periodic acoustic properties to temporal events well below the second formant^39^. Spectral representations were computed from FFRs using the fast Fourier transform (FFT). Specifically, for each participant and each stimulus condition, FFRs were averaged across trials, multiplied with a Hanning window, and an FFT was calculated. The nature of F1 encoding was quantified from the response FFTs, defined as the peak amplitude in the response spectra between 270 and 374 Hz, i.e., the expected F1 range from the stimuli. The location of these local maxima provided an estimate of the F1 frequency as encoded in the FFR. For each participant, the F1 estimate for each stimulus condition was correlated with the stimulus F1 values to compute an index—F1-encoding, to indicate how well the F1 differences between tokens were captured in the subcortical responses.

Vowel duration was estimated by how well the FFR tracked the trajectory of F0 in the stimuli. As mentioned above, phase-locking at the subcortical regions is limited to lower frequencies^39^, and we observed that in our current study, phase locking activity over time is most robust and consistent across stimulus conditions at the fundamental frequency level (see Appendix Fig. S1). To accurately estimate the neural tracking of VD, the duration of the F0 (which is also part of the vowel) was used as a proxy for VD, rather than that of the Formant structure. The ability of the FFR to follow F0 in the stimuli was evaluated using a periodicity detection short-term autocorrelation function^23,40^. Specifically, a sliding window analysis was applied to the FFRs in which a 40-ms bin was shifted in 1 ms steps to produce a total of 111 overlapping bins for FFRs to the short VD tokens and 311 bins for FFRs to the long VD tokens. Each of the time bins was cross-correlated with itself to determine how well the bin matched a time-shifted version of itself. The maximum (peak) autocorrelation value (between 0 and 1) was recorded for each bin, with higher values indicating more periodic time frames. For each stimulus and for each participant, the autocorrelation peaks (*r* values) from all the time bins were averaged to derive an autocorrelation-peak-average. We recorded time bins whose autocorrelation *r* values were larger than the autocorrelation-peak-average; then computed the time differences between the onset bin (i.e., the earliest time bin where autocorrelation* r* value was no less than the average) and offset bin (i.e., the last time bin where autocorrelation* r* value was no less than the average) to reflect the F0 time-course tracked by the FFR. The length of the F0 trajectory was then used to estimate the VD as encoded in the FFR. Similar to the F1-encoding, a VD-encoding index was also computed for each participant to indicate how well the VD differences between tokens were captured in the subcortical responses. Finally, the VD-encoding index was divided by the F1-encoding index to construct a neural counterpart of the cue-weight ratio, i.e., a cue-encoding ratio for each participant.

### Brain-behavior relationships

To examine whether and how subcortical encoding predicted behavioral cue weighting, multiple regression analysis was performed with the behavioral cue-weight ratio as the dependent variable, and the two neural indices (i.e., cue-encoding ratio and SRC) as the predictors. In addition to the neural predictors, years of musical training and self-rated L2 proficiency were also entered into the model to account for the potential influences of musical and L2 experiences.

## Supplementary Information

Supplementary Information.

## Data Availability

The experimental stimuli and the datasets generated during the current study, along with the analysis code, are available on Open Science Framework at http://doi.org/10.17605/OSF.IO/2ZR9W.
