# Supplementary material for: Individual variability in subcortical neural encoding shapes phonetic cue weighting
Source: Sci Rep. 2023 Jun 20;13:9991. doi: 10.1038/s41598-023-37212-y (PMC10281973; doi:10.1038/s41598-023-37212-y)
Supplement: Supplementary file 1 — Supplementary Information. [file 41598_2023_37212_MOESM1_ESM.pdf]

Title: Individual variability in subcortical neural encoding shapes phonetic cue weighting

Authors: Jinghua Ou*, Ming Xiang, & Alan C. L. Yu*

Department of Linguistics, University of Chicago, IL, USA

Supplementary Materials

Table S1. Musical and Language backgrounds for individuals in the final sample.

subject	Years of Musical training	Onset of musical training (age)	First language(s)	Other Languages and self-reported proficiencies (1-7, 1 as 'not proficient', 7 as 'very proficient') averaged for speaking and listening
S31	13	5	English	French (3.5)
S107	10	6	English	German (5.5)
S108	6	12	English	German (3)
S109	7	11	English	Polish (3.5)
S110	8	11	English	Mandarin (4)
S111	0	N/A	English	N/A
S112	6	9	English	Hindi (4)
S113	9	9	English	N/A
S114	0	N/A	English	Spanish (5.5); Portuguese (3.5)
S115	13	5	English	Spanish (6); French (5); German (5); Italian (4)
S116	14	5	English	Hebrew (5.5); French (3)
S117	7	7	English	Mandarin (6)
S118	4	9	English	Mandarin (4.5)
S119	11	5	English	Spanish (3)
S121	0	N/A	English	N/A
S123	4	8	English	N/A
S124	5	3	English; Japanese	Spanish (5)
S125	10	12	English	Korean (3)
S127	16	4	English	Vietnamese (3)
S128	7	9	English	Spanish (3)
S129	13	4	English	Urdu (4)
S131	4	8	English; Mandarin	Spanish (4.5)
S132	11	7	English	French (4)
S134	6	9	English; Hungarian	N/A
S135	3	9	English	Spanish (4.5)

Table S2. Formant frequencies of the vowel continuum.

		Formant Frequencies (Hz)		
	Stimulus Step	F1	F2	F3
/i/	1	270	2300	3019
	2	296	2243	2931
	3	322	2186	2843
	4	348	2129	2755
/ɪ/	5	374	2072	2667

Table S3. Results of the regression model for cue-weight ratio that included L2 proficiency, years of musical training and cue-encoding ratio as fixed effects.

Predictor	β	*SE*	*t*	*p*
Intercept	0.17	0.58	0.305	.764
L2 proficiency	-0.09	0.10	-0.92	.365
Musical training (yrs.)	-0.03	0.04	-0.66	.514
Cue-encoding ratio	1.09	0.20	5.45	< .001***

Supplementary analysis

Time frequency analysis (TFA) was performed on the EEG data to facilitate extraction of cue encoding of both Formant (F1) and Vowel Duration (VD) in the same computational method. The single-trial EEG were decomposed into time-frequency representations using a moving window Fast Fourier Transform approach implemented in Matlab. Specifically, the single-trial data were convolved using a Hanning-tappered 50 ms window that moved in steps of 10 ms along the temporal dimension. In the spectral dimension, a set of sinusoidal wavelets with linearly increased cycles from 3 cycles from the lowest frequency (60 Hz) to 15 cycles for the highest frequency (500 Hz) was used. For each time-frequency point in the stimulus window, the spectral power was divided by the average power in the baseline window (50 ms prior to stimulus onset) and transformed into a dB value. Fig. S1 demonstrates the time frequency representations based on the group-averaged FFRs. In each stimulus condition, we observed robust phase locking at around 100 Hz, which corresponds to the F0 of the stimuli.

Similar to the analytical procedures used in Section EEG data analysis, F1 encoding was defined as the peak of spectral power in the response spectrogram between 270 and 374 Hz. The location of the power maxima was used as an estimate of the F1 frequency as encoded in the FFR. For each participant, the F1 estimate for each stimulus condition was correlated with the stimulus F1 values to compute the F1-encoding index. To extract cue encoding of VD, a spectral-power-average at 100 Hz was computed by averaging the spectral power from all the time bins. We recorded time bins whose spectral power were larger than the spectral-power-average; then computed the time differences between the onset bin and offset bin to indicate the F0 time-course tracked by the FFR. Similar to the F1-encoding, a VD-encoding index was also

computed for each participant. Finally, the VD-encoding index was divided by the F1-encoding index to construct a cue-encoding ratio for each participant.

To examine brain and behavior relations, the cue-specific neural encoding indices were used to correlate with the behavioral cue weights, and the results demonstrated a significant correlation between VD-encoding and VD cue weights ($r = 0.43$, $p = 0.02$), while that between F1-encoding and Formant cue weight was marginally significant ($r = 0.34$, $p = 0.09$). We also correlated the cue-encoding ratio derived from the current TFA analysis with the behavioral cue-weight ratio, and observed a significant relationship between the two ($r = 0.68$, $p < 0.001$). The magnitudes of the correlations above were weaker in comparison to the correlations obtained when two different methods (i.e. FFT and autocorrelation) were used to estimate cue encodings (F1-encoding and Formant cue weight: $r = 0.61$; VD-encoding and VD cue weights: $r = 0.39$; cue-weight ratio and cue-encoding ratio: $r = 0.78$). This reduction in correlation magnitude could be attributed to the lower time/frequency resolution resulting from the trade-off between the two dimensions in TFA.

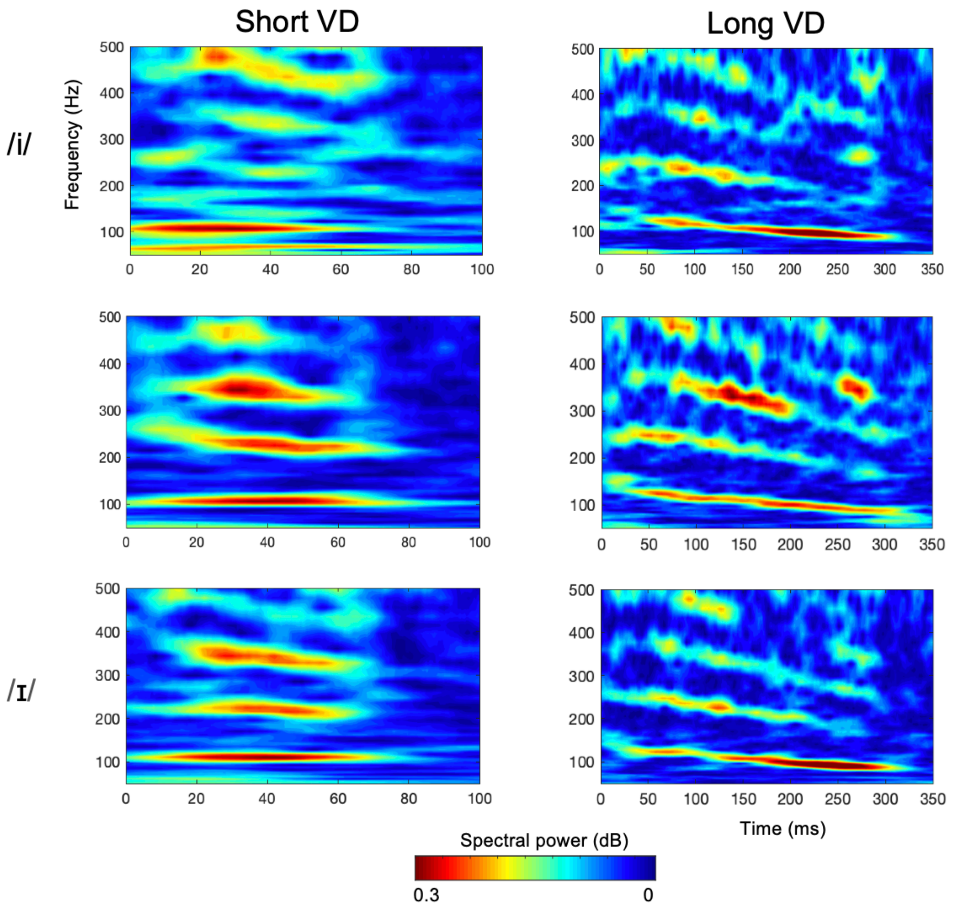

Fig. S1. Time frequency representations of the FFRs by three Formant (in rows) and two VD (in columns) conditions. Colors represent the spectral power, with warmer color indicating higher power.
